# An Unusual Presentation of Obstructive Jaundice Due to Dilated Proximal Small Bowel Loops After Gastrojejunostomy: Afferent Loop Syndrome

**DOI:** 10.7759/cureus.21258

**Published:** 2022-01-14

**Authors:** Mahrukh Ali, Om Parkash, Jehanzeb Shahid

**Affiliations:** 1 Gastroenterology and Hepatology, Aga Khan University Hospital, Karachi, PAK; 2 Radiology, Aga Khan University Hospital, Karachi, PAK

**Keywords:** gastric, rare, gastrojejunostomy, obstructive jaundice, afferent loop syndrome

## Abstract

Afferent loop syndrome is reported to be one of the very rare complications after gastrojejunostomy. The usual presentation in patients is with abdominal pain, distension and vomiting. It may present acutely because of complete obstruction, usually occurs early after surgery and is lethal in its course unless treated promptly with surgical management. In chronic cases obstruction is intermittent. There may be a reflux of bowel material into the biliary system and because of bacterial overgrowth patient may present with ascending cholangitis and obstructive jaundice. Here we report a case of 43-year-old gentleman presenting with jaundice, diffuse abdominal pain and distension. Later on, he was found to have a recurrence of gastric carcinoma associated with peritoneal carcinomatosis after subtotal gastrectomy and gastrojejunostomy for gastric carcinoma one year ago. He was diagnosed to be a case of afferent loop syndrome presenting as obstructive jaundice. The patient was managed conservatively by endoscopic decompression after confirmation of the diagnosis of afferent loop syndrome.

## Introduction

Afferent loop syndrome (ALS) is caused by the obstruction and dilatation of small intestinal loops proximal to the anastomosis leading to accumulation of biliary and pancreatic secretions and dilatation of these channels. It rarely presents as a complication of gastrectomy followed by gastrojejunostomy [1,2]. It may present after other procedures such as pancreaticoduodenectomy and few case series have been reported [3,4]. It is difficult to diagnose it clinically because of non-specific and vague symptoms [5,6]. Its management includes both surgical and non-surgical options. However, non-surgical management is preferred because of the ill health of the patients and that includes percutaneous and endoscopic approaches [3]. Here we report a case of ALS in a patient with gastric cancer who underwent gastrojejunostomy.

## Case presentation

A 43-year-old gentleman who was diagnosed as a case of signet cell carcinoma of the stomach and had undergone subtotal gastrectomy and loop gastrojejunostomy one year ago, presented with diffuse abdominal pain for one month, recent onset abdominal distention, vomiting, jaundice and absolute constipation. On physical examination, he was alert, vitally stable but was looking emaciated and icteric. The abdomen was distended with sluggish bowel sounds. The rest of the systemic examination was unremarkable. Based on the history and clinical examination the initial impression was disease recurrence and metastasis leading to intestinal obstruction.

**Figure 1:**
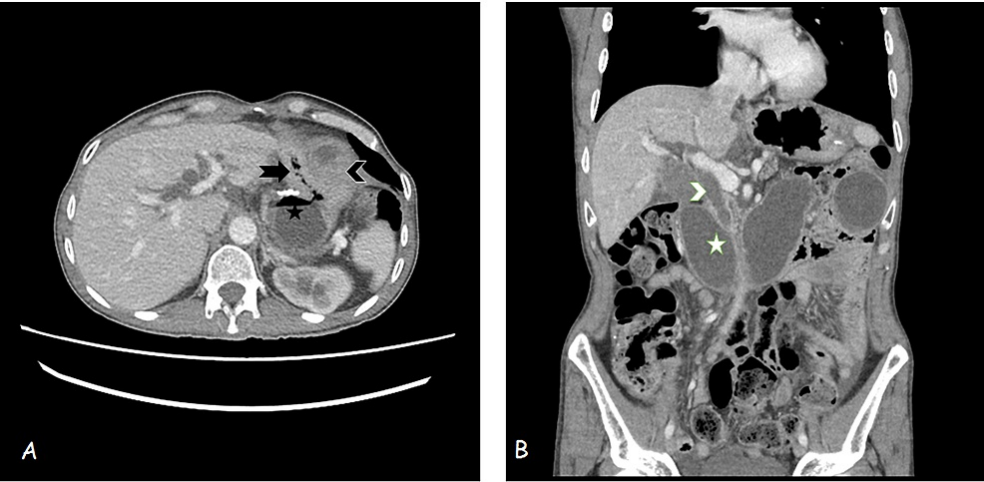
Contrast Enhanced CT Abdomen and Pelvis, Axial (A) and Coronal (B) Sections A - Axial section showing subtotal gastrectomy (black star) and loop gastro-jejunostomy. The efferent loop anastomotic site appears patent (black arrow) but the afferent loop anastomotic site shows thickening (black arrowhead). B - Coronal section shows dilated afferent jejunal loop (white star) in mid-abdomen with common bile duct (CBD) seen opening into that loop (white arrowhead). CBD is also appearing dilated with dilated intrahepatic biliary channels as well.

The laboratory investigations of the patient are shown in Table 1. Upper gastrointestinal endoscopy showed dilated and erythematous mucosa of the afferent limb. Biopsy of the anastomotic site showed no evidence of malignancy and jejunal biopsy showed numerous foci of poorly differentiated signet ring adenocarcinoma. Computed tomography of the abdomen revealed diffuse gastric wall thickening just lateral to the gastrojejunal anastomotic site, associated with peritoneal carcinomatosis. Dilatation of proximal small bowel loops causing reflux in intra- and extrahepatic biliary channels as shown in Figure 1. He was diagnosed as having Afferent Loop Syndrome and was managed conservatively.

**Table 1:** Laboratory Data of the Patient With Afferent Loop Syndrome

INVESTIGATIONS	REFERENCE VALUES	RESULTS
Serum Total Bilirubin	0.1-1.2 mg/dl	7.5 mg/dl
Serum Direct Bilirubin	0-0.2 mg/dl	5.6 mg/dl
Serum Indirect Bilirubin	0.1-0.8 mg/dl	1.9 mg/dl
Serum GGT (Gamma Glutamyl Transferase)	<55 IU/L	534 IU/L
ALT (Alanine Aminotransferase)	<45 IU/L	155 IU/L
Serum Alkaline Phosphatase	45-129 IU/L	296 IU/L
AST (Aspartate Aminotransferase)	<35 IU/L	85 IU/L
Hemoglobin	12.3-16.6 g/dl	13.8 g/dl
Hematocrit	38.4-50.7 %	42.1 %
WBC (white blood cells)	4.8-11.3 x 10^9^/L	4.3 x 10^9^/L
Platelets	154-433 x 10^9^/L	292 x 10^9^/L
Serum Sodium	136-145 mmol/L	134 mmol/L
Serum Potassium	3.5-5.1 mmol/L	3.9 mmol/L
Serum Chloride	98-107 mmol/L	93 mmol/L
Serum Bicarbonate	20-31 mmol/L	29.6 mmol/L
Plasma Prothrombin Time	9.1-13.1 seconds	10.9 seconds
Serum Creatinine	0.9-1.3 mg/dl	0.9 mg/dl

## Discussion

Afferent loop syndrome is defined as the dilatation of small bowel proximal to the anastomotic site of gastrojejunostomy [3] and is classified as acute and chronic based on the time of presentation after surgery, i.e., acute usually presents within a week of surgery as severe abdominal pain and vomiting while chronic takes many months or years of surgery and presents as subacute intestinal obstruction manifesting as abdominal distention that usually gets relieved with few episodes of vomiting. The afferent loop is usually compressed by post-surgical adhesions, stricture formation in the anastomotic site, recurrence of cancer and peritoneal carcinomatosis, all causing the Afferent Loop Syndrome [1-2]. In chronic afferent loop syndrome, the clinical symptoms are due to dilatation of the bowel due to raised intraluminal pressure followed by meals caused by biliary and pancreatic secretions.

This case is a type of chronic afferent loop syndrome presenting as obstructive jaundice because of disease recurrence and peritoneal carcinomatosis. The clinical symptoms and signs were suggestive of sub-acute intestinal obstruction and disease recurrence. CT scan done to look for the cause of obstruction revealed Afferent Loop Syndrome. Obstructive jaundice in such cases occurs when the pressure in the afferent loop is more than 18 cmH_2_O, leading to obstruction to biliary drainage and dilatation of biliary channels [7]. Cases of ascending cholangitis have been reported after pancreaticoduodenectomy [8]. Although the increase in afferent loop pressure affects both the bile duct and pancreas but pancreatitis incidence is less because of the protection offered by the Sphincter of Boyden [4]. There is no specific laboratory finding or X-ray findings helping in the diagnosis of the afferent loop syndrome, however abdominal ultrasonography and CT scan are very helpful for the diagnosis. Cholangiography has a diagnostic value when a CT scan is inconclusive [9].

The management includes surgical and non-surgical options [4,6]. The patients who are not stable enough for surgery, especially, those who are suffering from disease recurrence, non-surgical options include endoscopic decompression and percutaneous trans-hepatic catheter drainage [4,7]. In 75% of patients with afferent loop syndrome, surgical treatment is not successful because of the bad health condition of the patient and the spread of the disease [10,11]. Our patient was not a candidate for surgical management owing to his poor health condition, and recurrence and spread of carcinoma. However, endoscopic decompression was performed that lead to symptomatic relief.

## Conclusions

Afferent loop syndrome is a rare complication of gastrojejunostomy and in patients who presents with the sign and symptoms of intestinal obstruction and obstructive jaundice this condition should be considered in the differential diagnosis. Early recognition of this condition can lead to effective management and avoidance of complications.
